# Supplementary material for: Characterization of cytokine treatment on human pancreatic islets by top-down proteomics
Source: bioRxiv. 2025 May 15:2025.05.12.653562. Preprint. [Version 1] doi: 10.1101/2025.05.12.653562 (PMC12132588; doi:10.1101/2025.05.12.653562)
Supplement: Supplement 1 [file media-1.pdf]

Supplementary Information for

Characterization of cytokine treatment on human pancreatic islets by top-down proteomics

Ashley N Ives[1], Tyler Sagendorf[2], Lorenz Nierves[2], Tai-Tu Lin[2], Ercument Dirice[3], Rohit N Kulkarni[4-6], Ljiljana Paša-Tolić[1], Wei-Jun Qian[2*], James M Fulcher[1*]

[1]Environmental Molecular Sciences Laboratory, Pacific Northwest National Laboratory, Richland, WA 99354, USA

[2]Biological Sciences Division, Pacific Northwest National Laboratory, Richland, WA 99354, USA

[3]Department of Pharmacology and Medicine, New York Medical College School of Medicine, Valhalla, NY 10595, USA

[4]Islet Cell and Regenerative Biology, Joslin Diabetes Center, Boston, MA 02215, USA

[5]Department of Medicine, Beth Israel Deaconess Medical Center, Harvard Medical School, Boston, MA 02215, USA

[6]Harvard Stem Cell Institute, Harvard Medical School, Boston, MA 02215, USA

*Correspondence: weijun.qian@pnnl.gov (W.Q.) & james.fulcher@pnnl.gov (J.M.F.)

Contents

Table S1. Patient meta data. 3

Figure S1. Histogram of monoisotopic masses for observed proteoforms. 4

Figure S2. Histogram of relative standard deviations (RSD) based on label-free, intensity-based quantification of observed proteoforms. 5

Figure S3. Fragmentation (MS/MS) maps of select INS proteoforms. 6

Figure S4. Summary of top 30 most abundant CHGA and CHGB proteoforms. 7

Figure S5. Summary of top 30 most abundant SST proteoforms. 8

Figure S6. Fragmentation map for C-terminally amidated, oxidized GCG_{92-127}. 9

Figure S7. Proteoforms of HMGN1 and HMGN2. 10

Table S1. Patient meta data.
Age (years), sex, ethnicity, BMI, and HgbA1c (%) for donors.

Unique Identifier	Age (years)	Sex	Ethnicity	BMI	HgbA1c (%)
ADC5225A	27	M	Hispanic	30.7	5.2
ADC4231	29	M	White	32.5	5.5
ADIL276	51	F	Hispanic	30.1	NA
ADIR339	44	F	White	34.5	NA
ADH3495	53	F	Hispanic	33.3	6
ADH1303	50	F	White	20.3	NA

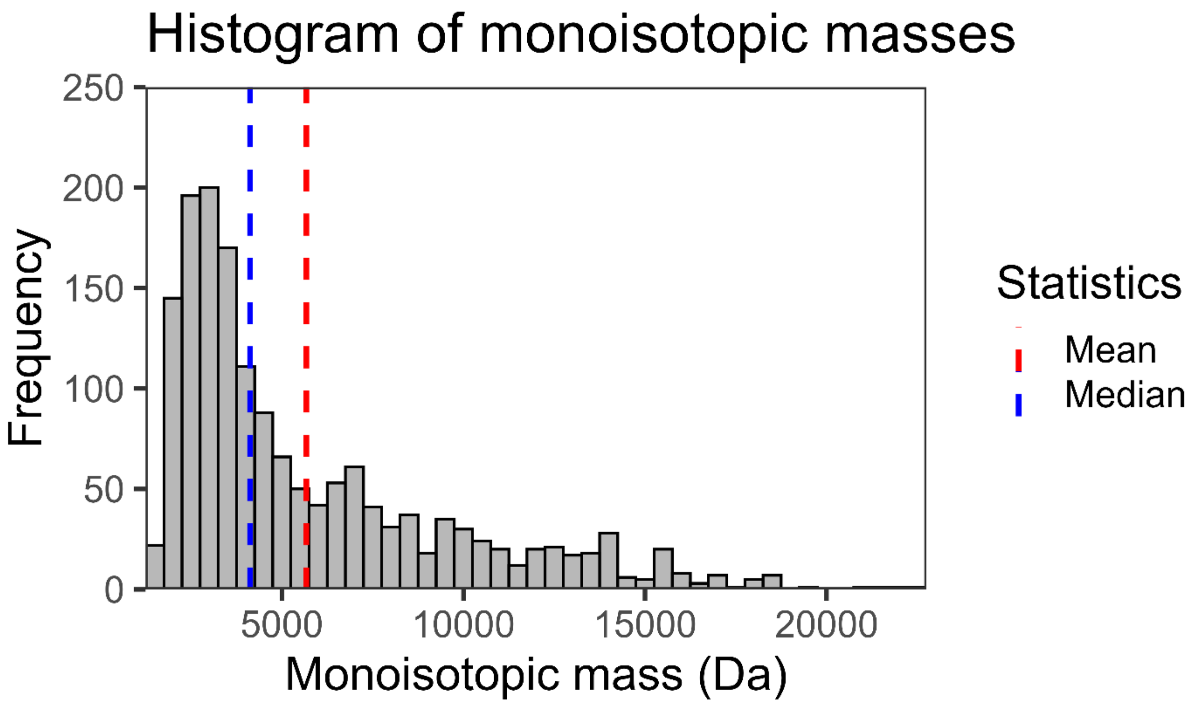

Figure S1. Histogram of monoisotopic masses for observed proteoforms.
The mean (red) and median (blue) are annotated as dashed lines.

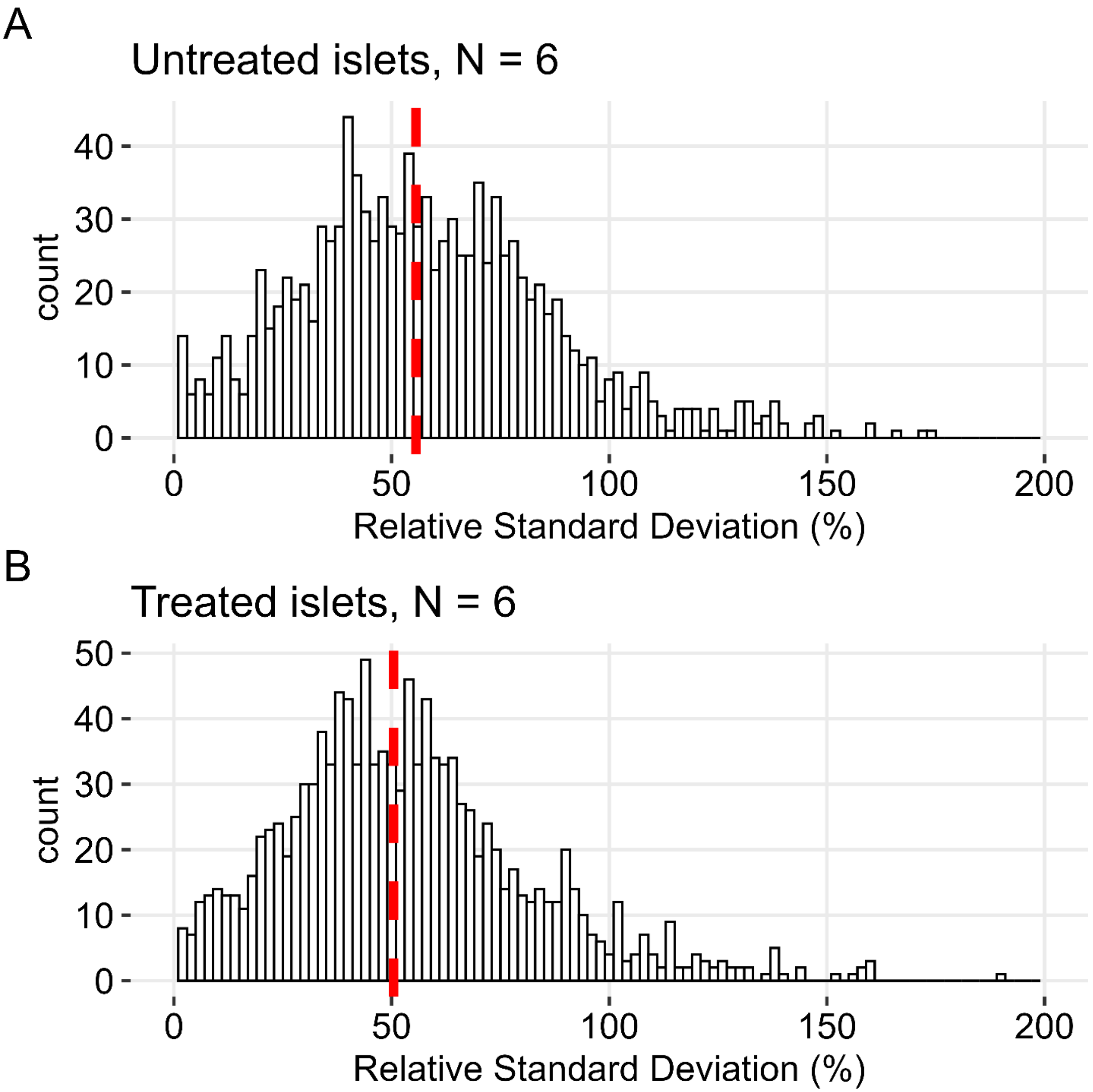

Figure S2. Histogram of relative standard deviations (RSD) based on label-free, intensity-based quantification of observed proteoforms.

RSDs are shown for (A) untreated, control islets and (B) islets after cytokine treatment following median normalization. The median (red) is annotated as a dashed line.

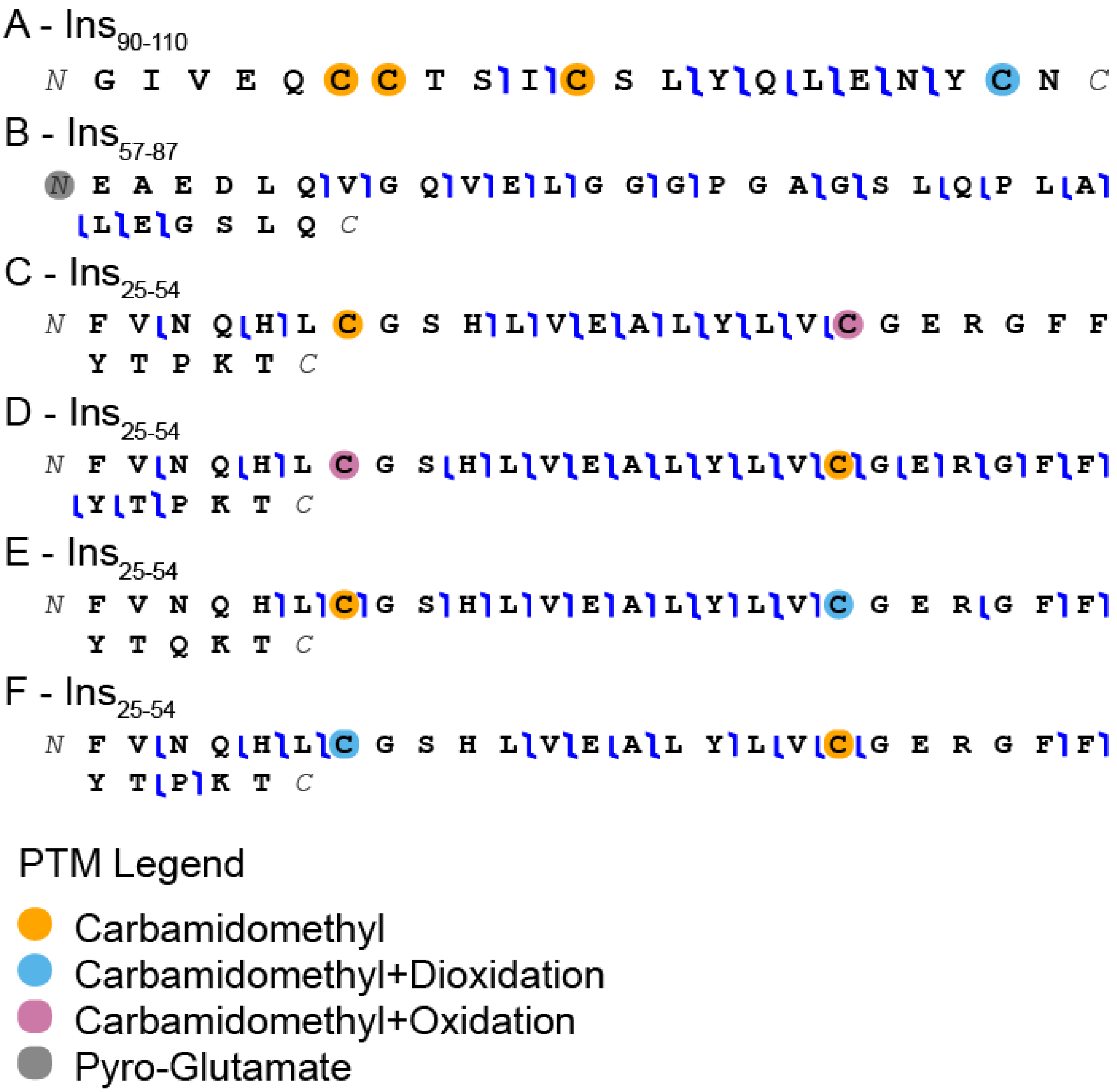

Figure S3. Fragmentation (MS/MS) maps of select INS proteoforms.

Maps denote matching fragment ions for searched candidate proteoforms including (A) dioxidation of Cys108, (B) pyro-glutamylation of the C peptide, oxidation within the B chain localized to either (C) Cys43 or (D) Cys31, and dioxidation within the B chain localized to either (E) Cys43 or (F) Cys31. Note that panels (C) and (D) are derived from the same proteoform spectral match scan. (E) and (F) are also derived from the same proteoform spectral match scan. Colored circles denote searched modifications including carbamidomethyl (gold), carbamidomethyl and dioxidation (blue), carbamidomethyl and oxidation (pink), and pyroglutamate (gray). Panel labels denote the first and last amino acid of a searched proteoform (i.e. $Ins_{firstAA\text{-}lastAA}$). Blue flags denote either *b*- (left-facing) or *y*-type (right-facing) fragment ions. The N- and C-termini are denoted as italized letters.

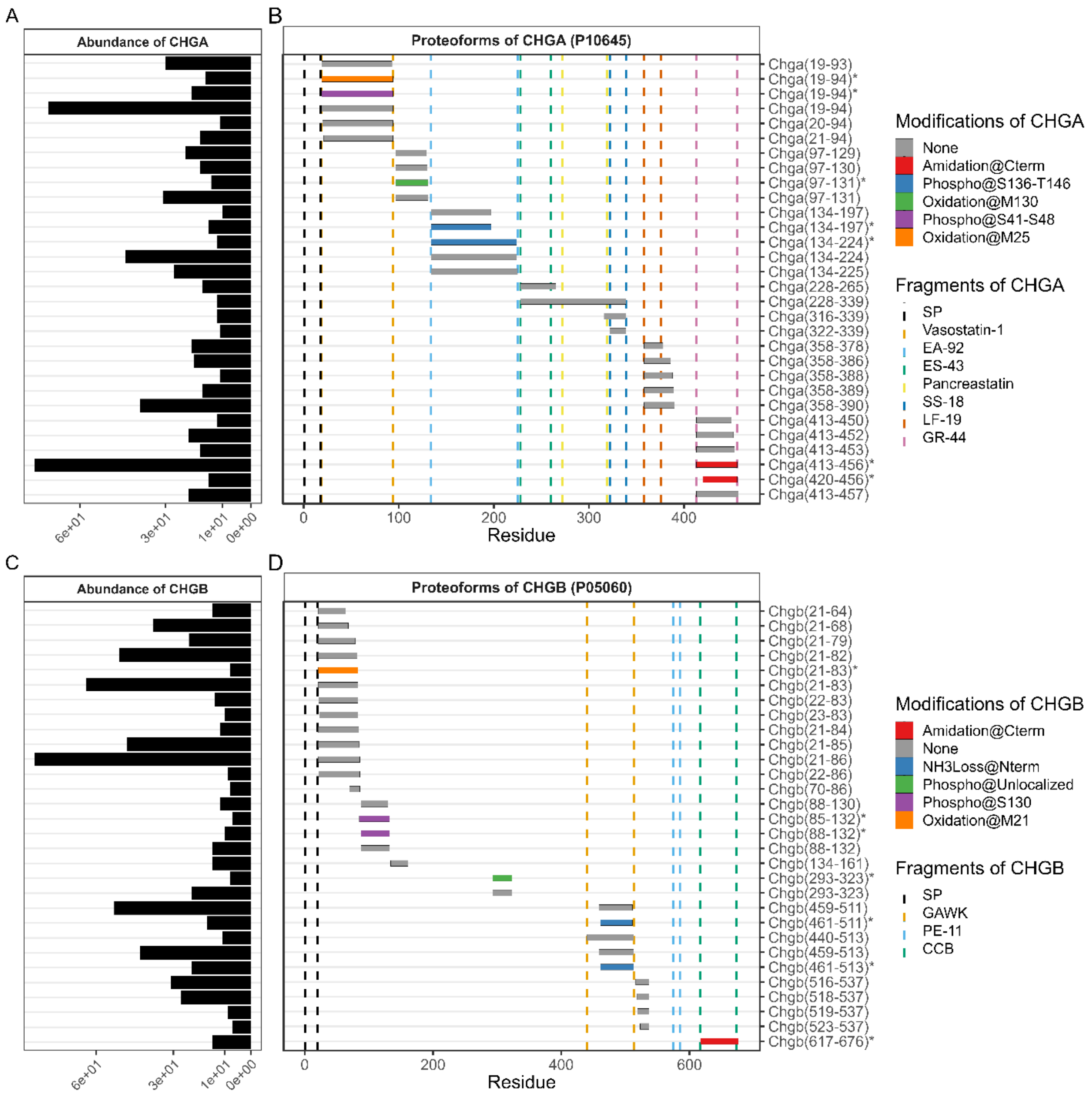

Figure S4. Summary of top 30 most abundant CHGA and CHGB proteoforms.
(A) Median spectral count abundance for (B) CHGA proteoforms. (C) Median spectral count abundance for (D) CHGB proteoforms. Rightmost panels map the first and last amino acid of a given proteoform (x-axis), and color fill denotes identified PTMs. Dashed vertical lines annotate the region of a given gene. Proteoforms are sorted top to bottom by ascending C-terminal amino acid ending position, followed by ascending N-terminal amino acid starting position. Y-axis labels denote the first and last amino acid of a given proteoform, and "*" is used to denote modified proteoforms.

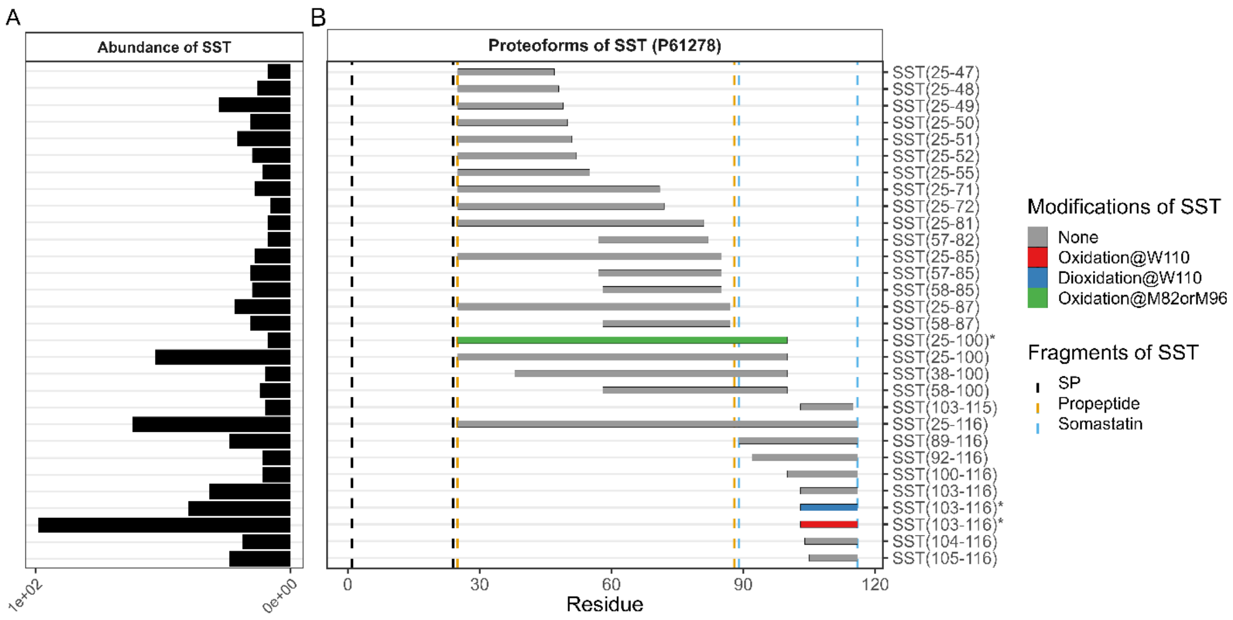

Figure S5. Summary of top 30 most abundant SST proteoforms.

(A) Median spectral count abundance for (B) SST proteoforms. Rightmost panels map the first and last amino acid of a given proteoform (x-axis), and color fill denotes identified PTMs. Dashed vertical lines annotate the region of a given gene. Proteoforms are sorted top to bottom by ascending C-terminal amino acid ending position, followed by ascending N-terminal amino acid starting position. Y-axis labels denote the first and last amino acid of a given proteoform, and "*" is used to denote modified proteoforms.

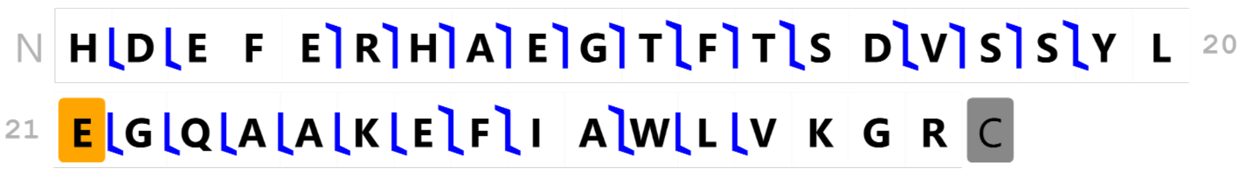

Figure S6. Fragmentation map for C-terminally amidated, oxidized $GCG_{92\text{-}127}$.
Assigned fragment ions are shown for the lowest E-value PrSM scan for C-terminally amidated, oxidized $GCG_{92\text{-}127}$. The amidation site is denoted with a gray box. The gold box denotes a carbonyl modification (+O, -2H). Blue flags denote either *b*- (left-facing) or *y*-type (right-facing) fragment ions. The N- and C-termini are denoted as gray letters.

A

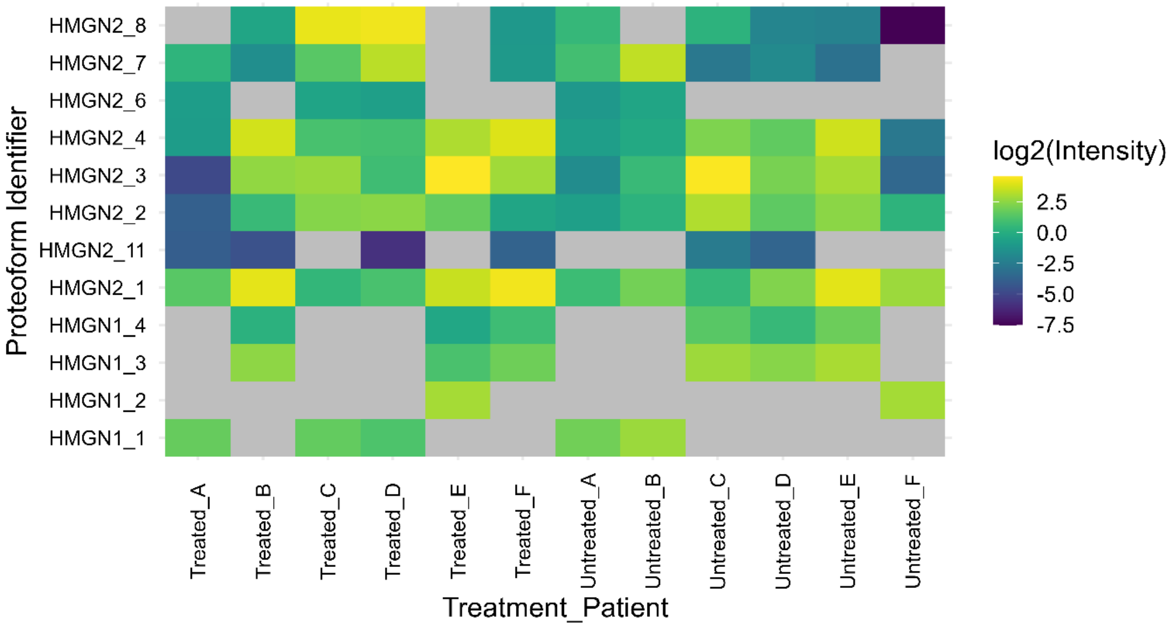

B

Identifier	Gene	Accession	firstAA	lastAA	Unexpected Modification
HMGN2_8	HMGN2	P05204	28	89	FALSE
HMGN2_7	HMGN2	P05204	25	89	FALSE
HMGN2_6	HMGN2	P05204	2	78	FALSE
HMGN2_4	HMGN2	P05204	25	90	FALSE
HMGN2_3	HMGN2	P05204	28	90	FALSE
HMGN2_2	HMGN2	P05204	43	90	TRUE
HMGN2_11	HMGN2	P05204	25	67	FALSE
HMGN2_1	HMGN2	P05204	2	90	FALSE
HMGN1_4	HMGN1	P05114	54	100	FALSE
HMGN1_3	HMGN1	P05114	24	100	FALSE
HMGN1_2	HMGN1	P05114	2	100	FALSE
HMGN1_1	HMGN1	P05114	2	100	TRUE

Figure S7. Proteoforms of HMGN1 and HMGN2.
(A) Heatmap of HMGN1 and HMGN2 proteoforms. Data is plotted as arbitrary proteoform identifiers (Gene_##) versus treatment_patient identifier. Fill denotes the median normalized log2(Intensity) as determined by label-free quantification. NA values are shown in gray. (B) Summarizes all proteoforms plotted in panel (A) including the first and last amino acid (firstAA, lastAA) based on the listed UniProt Accession. Proteoforms with unknown mass shifts are listed in column "Unexpected Modification".
